# The importance of including dynamic social networks when modeling epidemics of airborne infections: does increasing complexity increase accuracy?

**DOI:** 10.1186/1741-7015-9-88

**Published:** 2011-07-19

**Authors:** Sally Blower, Myong-Hyun Go

**Affiliations:** 1 Center for Biomedical Modeling, Semel Institute of Neuroscience & Human Behavior, David Geffen School of Medicine, University of California at Los Angeles, 10940 Wilshire Blvd, Suite 1450, Los Angeles, CA 90024, USA

## Abstract

Mathematical models are useful tools for understanding and predicting epidemics. A recent innovative modeling study by Stehle and colleagues addressed the issue of how complex models need to be to ensure accuracy. The authors collected data on face-to-face contacts during a two-day conference. They then constructed a series of dynamic social contact networks, each of which was used to model an epidemic generated by a fast-spreading airborne pathogen. Intriguingly, Stehle and colleagues found that increasing model complexity did not always increase accuracy. Specifically, the most detailed contact network and a simplified version of this network generated very similar results. These results are extremely interesting and require further exploration to determine their generalizability.

Please see related article BMC Medicine, 2011, 9:87

## 

Mathematical models have long been used to predict epidemic dynamics; in fact, models were first used over 200 years ago to study the effect of vaccination on smallpox epidemics [1]. Epidemic models can range from the very simple, consisting of only a few equations, to being so complex that they need to be simulated on supercomputers. Currently, a hotly debated issue in mathematical epidemiology is what degree of detail should be included in an epidemic model [2]. Simple models are built on few assumptions and are therefore more transparent; hence they can provide clear insights into the factors that drive an epidemic. However, if a model represents reality too simply, it is unlikely to be a useful tool for understanding and predicting epidemics. Complex models include many details and therefore may appear to be more "realistic" and accurate than simple models. However, complex models are based on many assumptions that are generally not evaluated to determine whether they are correct, and they can also include hundreds of parameters whose values are unknown or only imprecisely known. Consequently, complex models are not necessarily more accurate than simple models. The recent research by Stehle *et al*. [3] addressed the issue of simplicity versus complexity, and their results show that increasing the complexity of a model does not always increase accuracy.

Stehle *et al*. [3] performed an excellent and innovative study in which they first collected data on dynamic social contact networks and then used these data to model an epidemic generated by an airborne pathogen. They collected data over a two-day period from volunteers attending the 2009 Annual French Conference on Nosocomial Infections. Of the 1,200 attendees, 405 individuals participated in the study. For each study participant, Stehle *et al*. measured the number of social contacts (that is, face-to-face interactions) he or she had each day as well as the duration of each of these contacts. In total, 28,540 social contacts were recorded among the participants over the two days of the conference. Stehle and colleagues used this data set to construct networks. A network is specified in terms of nodes and edges (see Figure 1). In this case, a node represents a conference participant, and a social contact between two participants is represented by an edge. Figure 1 shows that the social contact network is different on day 2 than on day 1. A dynamic social contact network can be constructed by using the two static daily networks. Stehle *et al*. used radiofrequency identification (RFID) devices to collect their data. RFID is a short-distance interactive communication technology that allows devices to recognize each other within a small radius. Data were collected by having participants wear RFID devices embedded in their conference badges. The devices were set to a low threshold distance so that only proximate contacts were recorded. Contact information was collected at 20-second intervals. By using this innovative technology, it was possible to determine the number of social contacts for each participant in the study each day, to identify their social contacts, to measure the duration of each contact and to keep track of the order of these contacts. Contact data obtained in this manner are both exhaustive and free of the recall bias that plagues the alternative methods of recording contact information [4], such as asking study participants to keep diaries or collecting self-reported behavioral data through surveys [5,6]. As illustrated by Stehle *et al*. [3], the application of the RFID technology allows those who study infectious diseases to model transmission dynamics with unprecedented precision [7].

**Figure 1:**
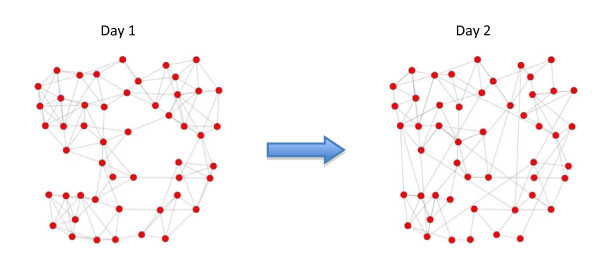
**Network data collected on two different days**. These two networks can be used to construct a dynamic social contact network. Red dots are nodes that represent study participants, and lines are edges that represent face-to-face contacts between study participants.

The main objective of the study conducted by Stehle *et al*. [3] was to determine how much information about the properties of the social contact network was necessary to accurately characterize an epidemic. They characterized epidemics in terms of the total number of infected individuals (that is, final size) and the number of days needed to reach the maximum incidence (that is, peak time). "Incidence" is defined as the number of individuals infected each day. Stehle *et al*. used their data set to construct a series of dynamic networks and then used these networks to simulate epidemics of two unspecified airborne pathogens. Both pathogens had similar biological properties, short incubation times (one to two days) and short infectious periods (two to four days). Networks were specified in terms of four properties: the number of contacts for each individual, the duration of contacts between participants, the order of contacts (that is, sequence) and the contact correlation. The value of the network's contact correlation was estimated by comparing network data collected on day 1 with network data collected on day 2. Specifically, they determined whether an individual who had a social contact with a particular individual on day 1 also had a contact with this individual on day 2. A total of nine networks were assessed, one of which was the observed network and the remaining eight of which were constructed by using the empirical data but varying the duration of contacts between participants, the order of contacts and the contact correlation. Unexpectedly, the results showed that even if the contact correlation is low in comparison with that of the observed network, the time needed for the epidemic to reach the maximum incidence is similar. However, if the contact correlation is much lower, the time required for the epidemic to reach the maximum incidence is considerably longer. Interestingly, Stehle *et al*. found that the network properties did not affect the final size of the epidemic if the attack rate (that is, the proportion of people infected by the end of the epidemic) was less than 10%. However, if the attack rate was greater than 10%, two properties of the network became important. They found that the lower the contact correlation, the larger the epidemic. In addition, the epidemic was even larger if the population average for the duration of contacts was used rather than the observed data for the duration of contacts between individuals. Notably, whether the network included the same order of contacts as observed in the data did not change the final size of the epidemic. The results of this study are new and surprising. They show that a more complex, detailed model may offer only a marginal improvement over simpler models that incorporate fewer, but key, details.

Stehle *et al*. [3] assessed the effects of dynamic social networks on the transmission of unspecified airborne pathogens with short incubation times and short infectious periods. The degree to which their results can be generalized to other pathogens and to other settings is not clear and should be the subject of future research. We suggest that it would be especially important to investigate other airborne pathogens that have longer incubation times and longer infectious periods, such as influenza, measles and tuberculosis; individuals with tuberculosis remain infectious for years. It would also be extremely interesting to use their modeling approach to try to determine the level of detail that must be included when modeling the transmission of sexually transmitted infections, such as HIV, using sexual contact networks [8]. Stehle *et al*. [3] have made a very important contribution to the modeling debate on simplicity versus complexity. Their results have significant implications for both data collection and model construction. They have shown that even if it is possible to collect very detailed data, it may not be necessary. Furthermore, they have shown that parsimonious models can sometimes be as good as complex models.

## Competing interests

The authors declare that they have no competing interests.

## Authors' contributions

MHG and SB reviewed the study and drafted the manuscript.

## Pre-publication history

The pre-publication history for this paper can be accessed here:

http://www.biomedcentral.com/1741-7015/9/88/prepub
